# Three-dimensional culture models of human endometrium for studying trophoblast-endometrium interaction during implantation

**DOI:** 10.1186/s12958-022-00973-8

**Published:** 2022-08-13

**Authors:** Xintong Li, Suranga P. Kodithuwakku, Rachel W. S. Chan, William S. B. Yeung, Yuanqing Yao, Ernest H. Y. Ng, Philip C. N. Chiu, Cheuk-Lun Lee

**Affiliations:** 1 Department of Obstetrics and Gynaecology, LKS Faculty of Medicine, The University of Hong Kong, Hong Kong S.A.R., China; 2 Department of Animal Science, Faculty of Agriculture, University of Peradeniya, Peradeniya, 20400 Sri Lanka; 3 Laboratory of Fertility Regulation, The University of Hong Kong Shenzhen Key, The University of Hong Kong-Shenzhen Hospital, Shenzhen, China

**Keywords:** Endometrium, Implantation, Placentation, 3D models, Organoid

## Abstract

During implantation, a symphony of interaction between the trophoblast originated from the trophectoderm of the implanting blastocyst and the endometrium leads to a successful pregnancy. Defective interaction between the trophoblast and endometrium often results in implantation failure, pregnancy loss, and a number of pregnancy complications. Owing to ethical concerns of using in vivo approaches to study human embryo implantation, various in vitro culture models of endometrium were established in the past decade ranging from two-dimensional cell-based to three-dimensional extracellular matrix (ECM)/tissue-based culture systems. Advanced organoid systems have also been established for recapitulation of different cellular components of the maternal–fetal interface, including the endometrial glandular organoids, trophoblast organoids and blastoids. However, there is no single ideal model to study the whole implantation process leaving more research to be done pursuing the establishment of a comprehensive in vitro model that can recapitulate the biology of trophoblast-endometrium interaction during early pregnancy. This would allow us to have better understanding of the physiological and pathological process of trophoblast-endometrium interaction during implantation.

## Background

In humans, pregnancy usually takes 39 weeks from embryo implantation to parturition. To establish a successful pregnancy, embryo attachment is the very first step and a synchrony between the receptive endometrium and the competent embryo is a must for the process. As a key event that determines the outcome of pregnancy, embryo implantation is tightly regulated by a plethora of factors from both the maternal side and the embryo side [1]. Owing to ethical considerations, the collection of human clinical samples, especially at the implantation site, is challenging. However, few studies on farm and laboratory animals have shed some light in this regard, even though the outcomes of the animal studies could not be fully adapted to human due to the physiological and anatomical specificity of human endometrium [2]. Therefore, it is a daunting task to study the interaction between the embryo and the endometrium in humans during pregnancy.

Defects in embryo implantation, placentation, and fetomaternal tolerance may result in pregnancy failures. Several disorders, including recurrent implantation failure, recurrent pregnancy loss, or pre-eclampsia (PE), are known to contribute to the pregnancy failure [3–5]. For majority of these diseases, the exact aetiology remains unknown due to the difficulties in mimicking the pathology in vitro, which makes it challenging for their diagnosis, treatment and prevention.

Several in vitro human culture models of endometrium have been developed to facilitate the study of the interaction between endometrium and embryo trophoblasts during implantation and post-implantation development. These models can be categorized into two-dimensional (2D) or three-dimensional (3D) cell/tissue culture models, and the 3D organoid models [6]. There is an increasing interest in the use of in vitro 3D models to simulate the physiological conditions, especially in corporation with the in vitro blastocyst and trophoblast surrogates.

There are many excellent reviews on the evolution and development of in vitro models of the endometrium [6–8]. In this review, the characteristics of the currently established 3D culture models of endometrium will be reviewed and evaluated. Their applications for the study of trophoblast-endometrium interactions upon the association with currently established embryo/trophoblast models under the in vitro conditions will be discussed, together with their potential implications for modelling of defective implantation and placentation. In comparison with other review articles [6–8] which mainly focus on either endometrial cell or trophoblast, our article is distinguished by its emphasis on the integrated 2D/3D system with both cell types for studying the maternal–fetal interaction.

## Trophoblast-endometrial interaction during implantation

### Embryo implantation

In a human menstrual cycle, pregnancy can only be established if implantation of an embryo happens within the window of implantation (WOI) [9–12]. Normally, the WOI occurs at the mid-secretory phase of the menstrual cycle, which generally happens 6 ~ 10 days after ovulation [13]. Several morphological and molecular signatures have been found to define the WOI, including decidualization of endometrial stromal cells, appearance of pinopodes, optimal development of endometrial glands and local production of cytokines and growth factors [12]. Together, the uterus is prepared for the implantation of embryo and establishment of pregnancy [14, 15]. In humans, implantation failure is a significant cause of infertility.

**Fig. 1:**
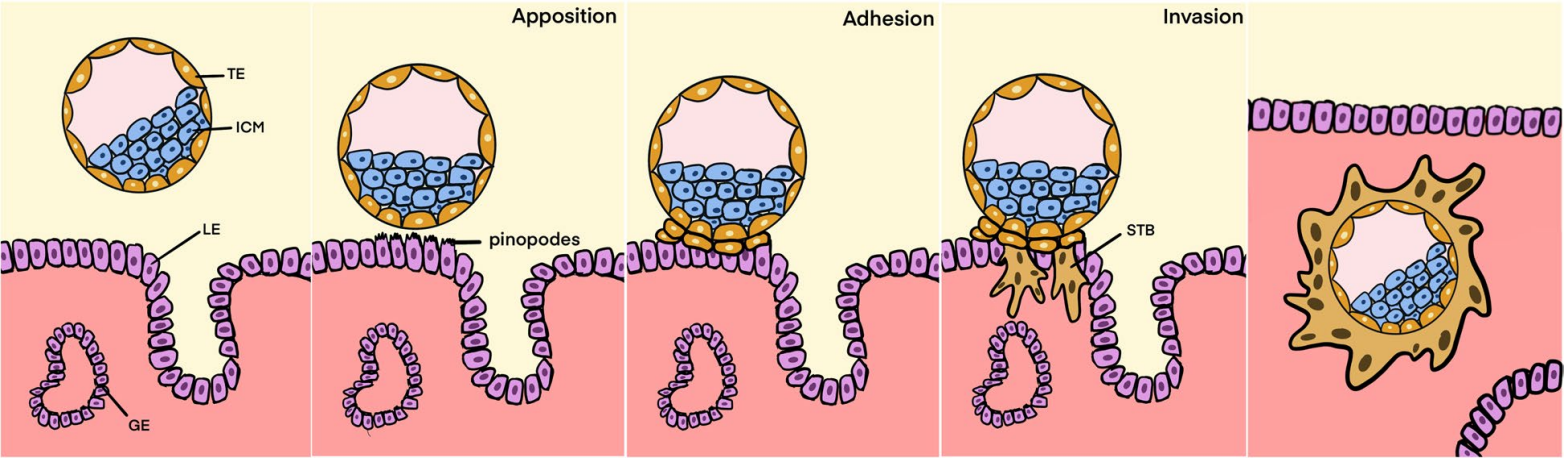
The process during the embryo implantation. The blastocyst implants to the maternal endometrium following sequential stages of apposition, adhesion and invasion. Each stage is associated with the production of molecular factors by both the blastocyst and the endometrium. ICM: inner cell mass, TE: trophectoderm, LE: luminal epithelium, GE: glandular epithelialium, STB: syncytiotrophoblast

The first three weeks of human pregnancy are generally considered as the implantation period, while placentation occurs from the third to twelfth week of gestation [16]. Embryo implantation marks the first direct cell-to-cell interaction between the mother and the developing embryo. It consists of a series of complicated and coordinated events. Implantation of the human embryo requires three sequential steps: apposition, adhesion and invasion (Fig. 1). During the secretory phase of the menstrual cycle, the endometrial stromal cells start to undergo decidualization under the drive of progesterone, converting the endometrial fibroblasts into the decidualized stromal cells, which facilitate invasion of trophoblasts of the blastocyst [17, 18]. In humans, blastocyst is formed from the fertilized zygote with the formation of inner cell mass (ICM) and trophectoderm (TE), which later migrates to the endometrium for the implantation after sequential cleavage and mitosis [19]. The orientated apposition allows interaction of the TE with the pinopodes (tiny microvilli protrusion) on the apical surface of endometrial luminal epithelium [1]. The blastocyst then adheres tightly to the endometrium, followed by invasion and penetration into the deeper stroma anchoring the embryo tightly in the endometrium [13, 20]. During this process, several molecular factors are produced from the maternal and the fetal sides working together to facilitate a successful implantation. These factors include steroid hormones, vasoactive factors, cytokines, growth factors, as well as the inter-cellular interaction [11, 21–25].

### Early placentation

**Fig. 2:**
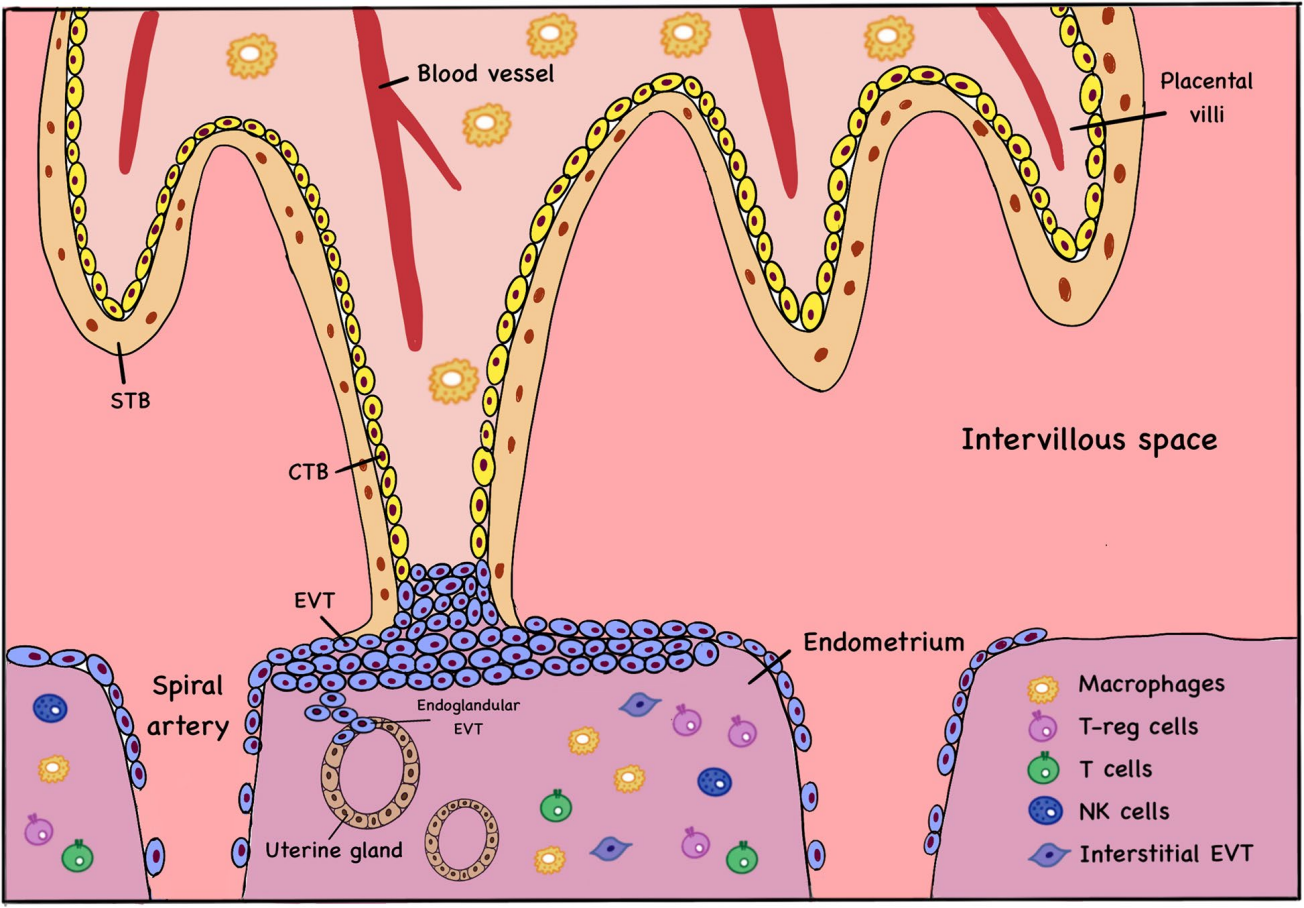
The maternal–fetal interface in early pregnancy. This is a simplistic drawing for the maternal–fetal interface. During early pregnancy, the anchored placental villi filled with trophoblast cell column interact with the maternal endometrium, forming the maternal–fetal interface. The inner cytotrophoblasts (CTB) fuse to form the outer syncytiotrophoblasts (STB), and the extravillous trophoblasts (EVT) invade the endometrium, uterine glands and the uterine spiral arteries. Maternal immune cells also participate in this process for maintenance of immune balance

After implantation occurs, extensive crosstalk takes place at the maternal–fetal interface, which is composed of the maternal decidua and the trophoblasts of the conceptus. A comprehensive schematic illustration of the constitutions of the maternal–fetal interface during early pregnancy is shown in Fig. 2. Throughout the course of pregnancy, the interface serves as the primary site for physical and functional interaction between the mother and the fetus, and is the site for placentation [26–30]. After embryo implantation, the placenta starts to develop from the third week of gestation, and the nutritional support of the embryo or fetus before the full establishment of the placenta tends to be histotrophic, i.e. originated from the secretion of decidual glands at the maternal–fetal interface. After the placenta is completely formed at the end of the first trimester, the fetus starts to receive full maternal blood supply [31, 32].

On the maternal side, the endometrial glands and the decidual stromal cells are capable to regulate the implantation activity and early embryo development via their secretory products [33–37]. Cytokines and growth factors, for example leukaemia inhibitory factors (LIF), are produced by the endometrial glands in the WOI, which can stimulate decidualization of stromal cells and prime implantation of blastocyst for attachment and adhesion [38–42]. In mice, the endometrial glands are capable to activate the blastocysts for initiation of implantation and acquisition of adhesion competence via the secreted glucose, amino acids (leucine and arginine) and proteins (osteopontin) [36, 43, 44]. The decidualized stromal cells promote the secretory function of the endometrial glands, mainly through the secretion of prolactin [45, 46]. Consistently, elevated expression of prolactin receptor has been identified in the endometrial glands both during the secretory phase of the menstrual cycle and during early pregnancy [45, 47, 48]. On the fetal side, the decidualized stromal cells regulate the invasion of trophoblasts [49]. The cells produce both pro-invasive factors (Interleukin-1β (IL-1β), IL-6, IL-11) and anti-invasive factors (IL-10, Vascular Endothelial Growth Factor (VEGF)), which together maintain the invasion of trophoblasts at a balanced state [50–53]. In addition, the maternal immune cells (e.g. decidual natural killer (dNK) cells, macrophages and leukocytes) with immune-modulatory functions to prevent immune rejection of the fetus also contribute to the organization of the maternal–fetal interface in early pregnancy [17, 54, 55]. The involvement of decidual immune cells during early pregnancy has been extensively studied [55–57].

On the fetal side, the TE develops into cytotrophoblastic shell [30]. Cytotrophoblasts (CTB) originate from the shell differentiated into the villous CTBs and the extravillous cytotrophoblasts (EVTs). The villous CTBs fuse to form the multinucleated syncytiotrophoblasts (STBs) with the hormone-secreting function, while the EVTs invade the decidua to the spiral arteries during the first trimester of pregnancy. When the EVTs reach the spiral arteries, they induce apoptosis of vascular smooth muscle and endothelial cells and replace the endothelium by fibrinoid material. The remodeling process transforms the spiral arteries to low-resistance, high-flow vessels ensuring sufficient fetal-maternal exchange [31, 58, 59]. Some studies have also reported the invasion of EVTs into the endometrial glands during early-first trimester. By migrating towards the endometrial glands and replacing the glandular epithelial cells, these EVTs may facilitate the endometrial gland secretion and the placental development [30, 60–63]. In sum, all these compartments exert important functions on the other compartments during the process of implantation and early pregnancy.

## In vitro culture models of human endometrium

Even though human pregnancy has been extensively studied, the molecular processes happening at the maternal–fetal interface during early pregnancy remain largely unknown. Due to the inherent ethical limitations of conducting in vivo studies using human subjects and other issues related in extrapolating the animal data to human situations [64], the in vitro culture models of human endometrium based on isolated primary cells are employed to mimic the implantation and early pregnancy process for investigation of normal and complicated pregnancy. Besides, the models will pave the way to initially test and validate the remedial/therapeutic measures in translational medicine.

### In vitro 2D models of human endometrium

At the very beginning of the development of in vitro culture models, endometrial cells were cultured in the 2D format. In these 2D models, endometrial tissues from donors were digested into single-cell suspension by methods such as filtration, cell adhesion, density gradient centrifugation, immunomagnetic selection and fluorescence-activated cell sorting to allow the separation of epithelial cells, stromal cells, endothelial cells and leukocytes. These cells were then cultured in a monolayer on culture plates under their own specific culture conditions [6, 65–67]. The main disadvantage of the 2D culture of primary endometrial cells is that they show reduced biological activities after several passages and diminished response towards sex hormones, which are not supportive to study their morphological and functional roles [6, 68]. Given the limited availability of primary human endometrial tissues, many researchers used endometrial adenocarcinoma and immortalized epithelial cell lines in 2D models to study the endometrial epithelial functions. However, even though the cell line models are relatively easy to access and maintain, the potential genetic aberration associated with prolonged culture can be an obstacle for investigating the physiological properties of endometrium [68].

The omission of cell-extracellular matrix (ECM) interaction is another major drawback of 2D culture models. ECM is the 3D matrix scaffold surrounding the cells. The endometrial ECM provides biochemical and biophysical support to the endometrial cells and plays important roles in the menstrual cycle and embryo implantation [6, 69]. A monolayer culture without ECM alters the functional activities of the endometrial epithelial cells; they lose their polarities and change their secretory functions [69]. Meanwhile, monolayers of multiple cell types are difficult to be co-cultured, thus unable to recapitulate the in vivo crosstalk between the endometrial stromal and epithelial cells. To summarize, the 2D culture models of endometrium provide the basis for development of the in vitro culture systems. However, they need to be further optimized for better recapitulation of the human endometrial physiology.

### In vitro 3D culture models of human endometrium

Since the 1980s, in vitro 3D culture models of the human endometrium have been gradually evolved as alternatives for the classic 2D culture models. These culture models have benefited in studies of early pregnancy mechanisms including embryo implantation, disease modelling and drug development [6]. Compared to the classical 2D culture models, the 3D culture models of the endometrium usually integrate the ECM plus multiple cell types into the culture system by encompassing the primary tissues/cells and cells from established cell lines in collagen, Matrigel, fibrin-agarose gel, and synthetic scaffolds for the better mimicking of the physiological structure [6, 70–72].

#### Cell-based 3D culture models of human endometrium

**Fig. 3:**
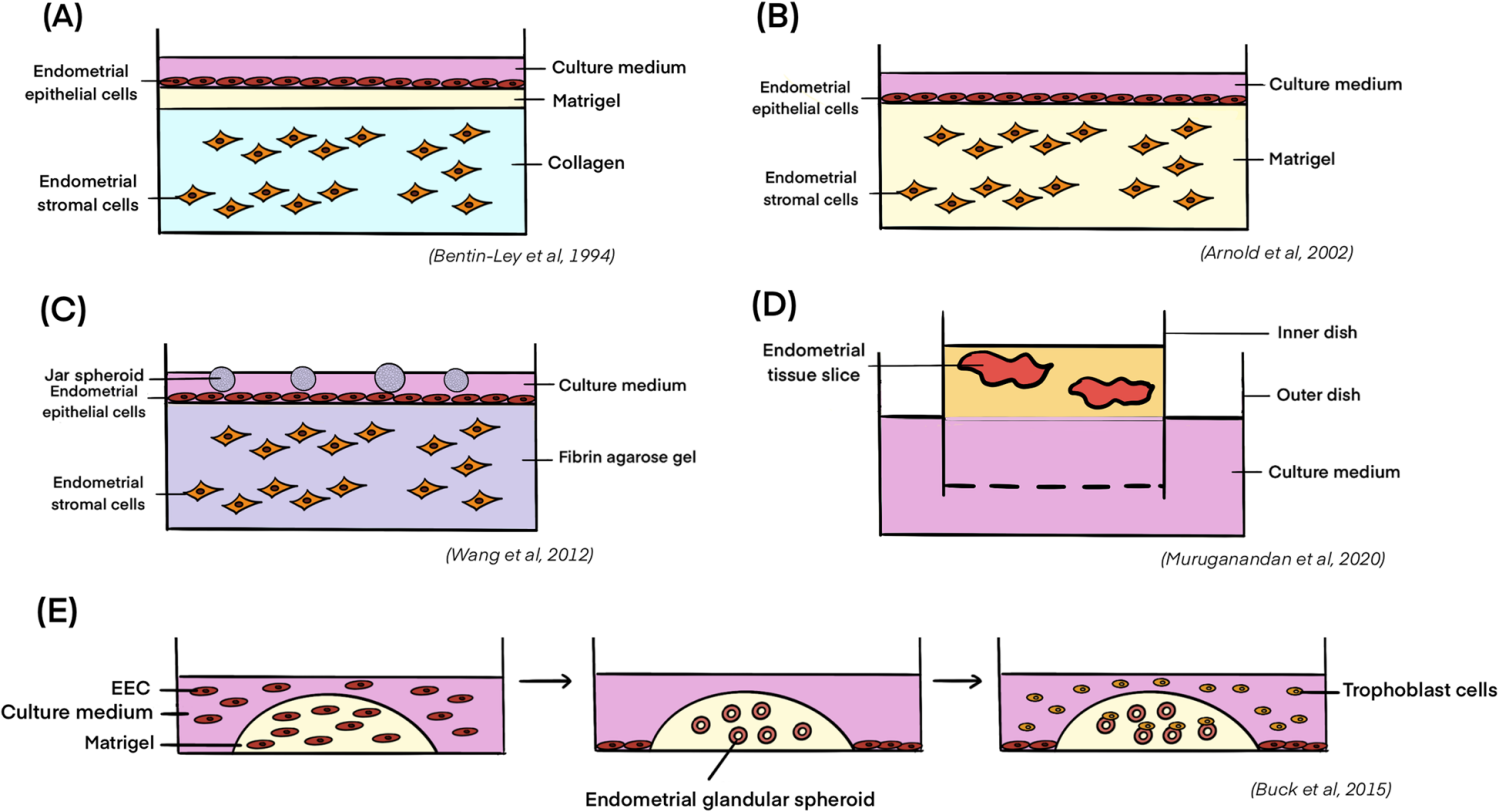
The established in vitro 3D culture models of endometrium. **A** Stromal cells were suspended in collagen followed by overlay of Matrigel and epithelial cells on top; **B** Stromal cells were resuspended in Matrigel, while epithelial cells (Ishikawa cells) were seeded directly on top; **C** Endometrial stromal cells were resuspended in fibrin-agarose gel followed by overlay of epithelial cells. JAR cell-derived spheroids were used as the trophoblast surrogates in the co-culture system; **D** Combined 2D/3D endometrial model which integrated the 3D endometrial glandular spheroids and the 2D monolayer of the endometrial epithelial cell lines, followed by addition of the trophoblasts for study of trophoblast invasion. **E** 3D culture model of endometrium using the human endometrial slices suspended in collagen matrix in the double-dish culture plate

At present, in vitro 3D culture models comprising the epithelial, stromal, endothelial and immune cells have been gradually established. The initial 3D culture models are simple and comprise of the endometrial glandular epithelial cells and the endometrial stromal cells [70]. In the model by Bentin-Ley et al. (Fig. 3A), endometrial glandular and stromal cells were isolated separately from endometrial biopsies by enzymatic digestion and sequential sedimentation. Collagen I was used as the ECM to embed the endometrial stromal cells, followed by overlay of a thin layer of Matrigel on the top surface. The isolated glandular epithelial cells were then seeded on the Matrigel layer. During culture of the 3D model in a medium supplemented with estrogen and progesterone, polarized development of the endometrial epithelial cells was achieved, with prominent glandular formation and presence of pinopode structure. The study clearly demonstrated the importance of ECM in the in vitro culture system and suggested potential paracrine interactions between the stromal cells and the epithelial cells of the endometrium.

An improved version of the 3D endometrial model was established later for examination of the impact of two contraceptive drugs, levonorgestrel and mifepristone, on expression of endometrial receptivity markers [73, 74]. The model demonstrated that the epithelial and the stromal cells expressed estrogen receptor (ER), progesterone receptor (PR) and several endometrial receptivity markers including LIF, IL-1β, VEGF and cyclooxygenase-2 (COX2) upon treatment with 0.3 nM of estrogen and 900 nM of progesterone. Levonorgestrel had no significant impact on the expression of the receptivity markers during progesterone stimulation, while mifepristone inhibited the expression of VEGF, Mucin-1 (MUC-1) and integrin α_V_β_3_ similar to that observed in vivo. Most importantly, human embryos can be incorporated to interact with the established 3D endometrial model, enabling the use of the model to study embryo implantation as well [73].

Another 3D culture model of the human endometrium utilized adenocarcinoma cell lines and Matrigel as the sole ECM support [71]. In this study, primary endometrial stromal cells were cultured in Matrigel, and Ishikawa cells, a cell line derived from endometrial adenocarcinoma cells (cancer cells of endometrial glandular origin) expressing epithelial-like phenotype, were seeded directly on top of the Matrigel (Fig. 3B). Differently from the Bentin-Ley’s study [70], this co-culture model allowed study of the impact of direct contact between the endometrial stromal cells and the glandular epithelial cells. Co-culture with the stromal cells triggered the production of glycodelin from the Ishikawa cells. Glycodelin is a major endometrial gland-derived glycoprotein produced in the secretory phase of menstrual cycle [75, 76]. The production of glycodelin indicates differentiation of the secretory glandular cells in vitro. The study further showed that the regulatory functions of stromal cell-derived secretory factors on the Ishikawa cells were reduced in the absence of the Matrigel. Thus, it was concluded that secretory factors from the stromal cells exerted paracrine actions regulating proliferation and differentiation of the co-cultured endometrial epithelial cells, and that Matrigel helped the cells function in more physiological manner in the 3D culture model. With the use of the co-culture model and treatment with spent medium from stromal cells cultured in conventional monolayer or Matrigel, it was demonstrated that both the stromal-factors and the ECM contributed to the in vitro biological activities of endometrial epithelial cells. One limitation of the study was that the adenocarcinoma Ishikawa cells instead of primary cells were used as the epithelial cell surrogates. Integration of the primary epithelial and stromal cells from the same patient should better recapitulate the physiological conditions of the human endometrium.

The conventional ECM materials, collagen and Matrigel, are easily degraded or shrunk in culture, which reduces the longevity of the co-culture. In order to address the problem, another endometrium-like in vitro 3D model has been established [72] utilizing agarose and fibrin as the ECM. Compounds including calcium chloride and tranexamic acid were supplemented to prevent the degradation of the ECM. In this model, the endometrial stromal cells were cultured in the supplemented fibrin-agarose, and the epithelial cells were seeded on top of the gel (Fig. 3C). Both the immortalized cell lines (Ishikawa cells and human endometrial stromal cells) and the primary stromal and epithelial cells from healthy fertile women were tested to construct the 3D model. Immunohistochemistry of specific cell markers suggested good viability and functionality of the co-cultured cells. With the fibrin agarose scaffold, the cells could be kept for at least 10 days with intact structure, outperforming the previously established 3D endometrial models. The integration of trophoblast spheroids into the model further allows a comprehensive investigation of the dynamic trophoblast invasion into the maternal endometrium, which will be reviewed in subsequent sections.

#### Combined 2D/3D culture models of human endometrium

To better understand the functions and contributions of the endometrial glands during early pregnancy, another in vitro combined 2D/3D culture model of human endometrium was established in 2015 [77]. In this study, three endometrial epithelial cancer cell lines, including HEC-1 cells, Ishikawa cells and RL95-2 cells, were first seeded into the Matrigel at a 1:1 ratio with the culture medium and allowed to solidify, followed by overlay of the same cell type in the form of single-cell suspension. The endometrial epithelial cells in the Matrigel formed 3D spheroid-like structure that recapitulate the endometrial glandular structure, while the cells in suspension settled and grew with classic 2D morphology on the bottom of the culture plate (Fig. 3E). Interestingly, only two of the endometrial cell lines, HEC-1 cells and Ishikawa cells, formed the spheroid-like structure with a lumen, while spheroids from RL95-2 cells did not show a clearly defined lumen and showed reduced expression of tight junction marker ZO-1. The model can be used to study the impact of epithelial junction on trophoblast-endometrium interaction in a 3D manner.

#### Tissue-based 3D culture model of human endometrium

Besides the cell-based 3D culture models, the culture of biopsied endometrial slice was attempted in the collagen I matrix using a double-dish culture method (Fig. 3D) [78]. The endometrial slice in the matrix exhibited hormone responsiveness in terms of increased expression of ER and PR in response to estrogen and progesterone stimulation, as well as decidualization of the stromal cells in response to decidualization inducers. The model demonstrated the importance of ECM in preserving the functionality of endometrium in vitro. Compared to the conventional cell-based models, which generally show diminished cell viability and hormone responsiveness in vitro approximately after 5 days, the endometrial tissue slice in this double-dish tissue-based model was viable after 21 days, which may benefit the studies on the turnover and cyclic remodelling of the endometrium.

Until now, several 3D in vitro culture models have been established and usually involve the endometrial stromal cells and either the luminal or glandular epithelial cells. However, there is no characterization of the glandular structure in these models [6]. Due to the interactive impact of the endometrial glands to other endometrial cell types especially during early pregnancy, the glandular structure should be incorporated into the 3D model of the endometrium in future for better study of the female reproductive biology.

#### Endometrial glandular organoid culture model

To study the endometrial glands, endometrial glandular organoids have been established as a novel in vitro 3D culture model [79–81]. Organoids are defined as ‘the self-organizing and genetically stable 3D culture systems, which recapitulate the molecular signature of the tissue of origin, and contain both the progenitor and the differentiated adult cells of the tissue of interest’ [82]. The endometrial glandular organoids have been regarded as an important tool for the study of female reproductive biology. Table 1 compares the properties of 2D, 3D and organoids in vitro culture models. Compared to the conventional 2D and 3D culture models, organoids contain multiple cell types within the intact 3D structure, together with the presence of ECM that mimic the physiological microenvironment. Unlike most of the endometrial 3D culture models that could only be cultured for less than 10 days, the organoids are stable to be cultured and passaged for a much longer time together with high genetic stability. Thus, the organoid culture model has been gradually applied for the modelling of development, diseases and testing/screening of drugs in regenerative medicine [83].

**Table 1:** Comparison of 2D, 3D and 3D organoid culture models in studies

	**2D model**	**3D model**	**3D organoid model**
Physiological recapitulation	Relatively low	Relatively high recapitulation but may be artificial	Highly recapitulate the tissue of origin
Cell-ECM interaction	Lack of interaction	Mimic Natural ECM environment	Mimic Natural ECM environment
Cell types	Usually only one cell type involved	Multiple cell types involved	Multiple cell types involved
Stability of the culture system	Very stable for cell lines but relatively less stable for primary cells	Usually not stable for a long time	Stable culture system
Ease of setup/cost	Easy to set up. Low cost	Set up requires high expertise. Relatively high cost	Specific culture medium required. Very high cost

Historically, Rinehart and colleagues developed a 3D model for culture of endometrial glandular epithelium in 1988 [84]. In this study, the endometrial glands were isolated from the endometrial tissue and embedded in Matrigel at the ratio of 1:1 with culture medium. The endometrial gland fragments initially spread in the Matrigel as monolayers of tiny colonies and eventually formed gland-like structure. Similarly, some other studies also found the development of prominent glandular structure from endometrial epithelial cells when they were cultured on basement membrane extracts (BME) or Matrigel. These cells showed polarization, hormone responsiveness and secretory functions that recapiculate the endometrial glands in vivo [85–88]. Even though the gland-like structures were not well characterized, the study set the basis for the development of the endometrial glandular organoid model after nearly 40 years.

**Fig. 4:**
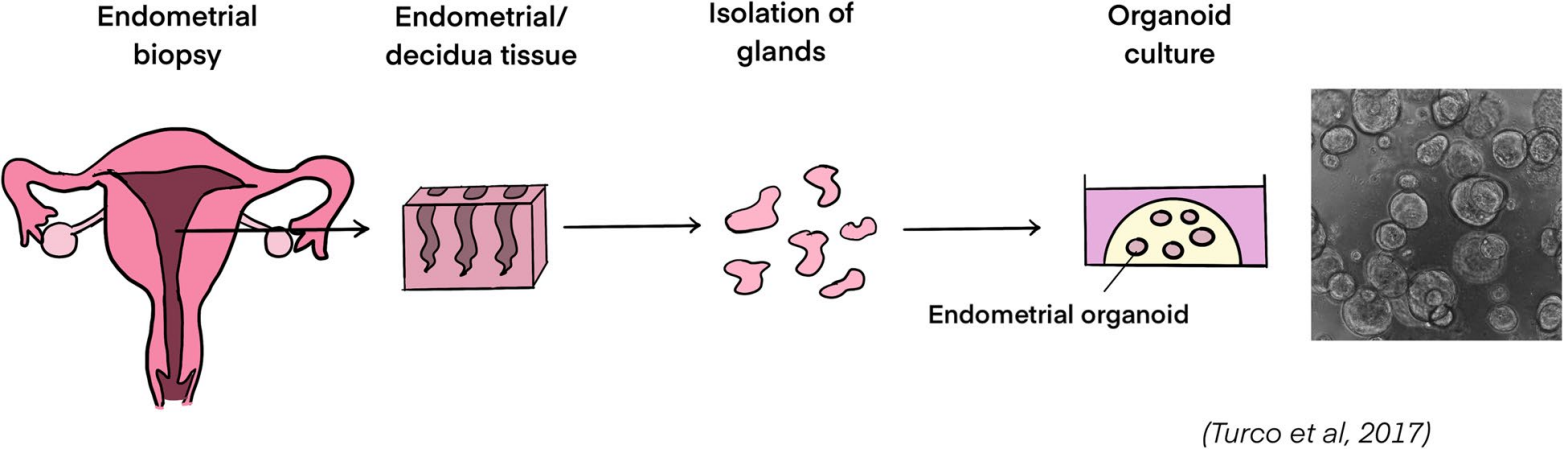
Establishment of endometrial glandular organoids from the primary endometrial tissue. Endometrial or decidual tissue is cut from the endometrial biopsy, followed by enzymatic digestion for the isolation of endometrial glands. The glandular cells will then be seeded in Matrigel for the formation of endometrial glandular organoids

Endometrial glandular organoids were derived from primary endometrial tissue from healthy non-pregnant endometrial tissue, pregnant decidua tissue, post-menopausal endometrial tissue and from tissue in diseased conditions including endometrial carcinoma and endometriosis tissues [80, 89]. To produce the organoids, the collected endometrial or decidua tissue biopsy was digested to isolate the endometrial glands, which were then suspended in Matrigel for 3D culture (Fig. 4) [80]. Specific expansion medium containing activin receptor-like kinase inhibitor A83-01, Fibroblast Growth Factor (FGF) 10, Epidermal Growth Factor (EGF), Hepatocyte Growth Factor (HGF), R-spondin-1, Noggin and nicotinamide had to be used for the culture of endometrial glandular organoids [80]. Under such condition, the endometrial glandular organoids could be established in 7 to 14 days from single epithelial cells, suggesting the presence of stem/progenitor cells [80]. The resulting organoids exhibited the classic epithelial-derived organoid structure with a clearly defined lumen. They could be maintained and expanded for more than six months with regular passaging, as well as cryopreservation and thawing for recovery [80, 81]. Molecularly, the endometrial glandular organoids expressed the classic glandular epithelial markers, including Cadherin 1 (CDH1), MUC-1, Forkhead Box A2 (FOXA2), Paired Box 8 (PAX8) and SRY-Box Transcription Factor 17 (SOX17). Their transcriptome was similar to that of the primary glands from normal endometria, confirming the glandular origins of the organoids [80, 81].

Physiologically, the endometrium responds to the cyclic change of estrogen and progesterone in terms of proliferation and differentiation of the endometrial glands, as well as spatio-temporal expression of the ERα and PR [80, 81]. Without the steroid hormones, the endometrial glandular organoids generally exhibited low expression of ERα and extremely low level of PR expression. Upon treatment with estrogen for 2 days followed by the co-treatment with progesterone for another 4 days, a significantly higher expression of both ERα and PR was found, which resembled the in vivo condition [80, 81]. Single-cell RNA sequencing revealed a higher number of ciliated cells, epithelial cells and secretory cells in the endometrial glandular organoids treated with estrogen and medroxyprogesterone acetate compared to the ones in the basal expansion medium, while the number of the unciliated cells and the stem/progenitor cells was reduced [81]. These features demonstrated similarities of the dynamic hormone responsiveness of the endometrial glandular organoids with the human endometrium in vivo.

The secretory function of the endometrial glandular organoids in vitro was similar to that of the endometrial glands during the menstrual cycle and early pregnancy. Pseudostratified columnar epithelial cells formed the outer layer of the endometrial glandular organoids and lined the cystic structure of the organoids [81]. The columnar epithelial cells exhibited prominent apical-basal polarity with abundant microvilli on the apical surface and had shown the evidence of the secretory function including the presence of secretory vesicles in the cytoplasm and the existence of glycogen as the major secretory product of endometrial glands. Upon exposure to the estrogen/progesterone supplemented-differentiation medium in presence of the secretory product of decidualized stromal cell, prolactin, and placental hormones, human chorionic gonadotropin (hCG) and placental lactogen for 8 days, the organoids developed the phenotype of decidual-glands similar to that in early pregnancy [80]. Collectively, the molecular signature and functional properties of the endometrial glandular organoids render them to recapitulate the physiology and pathology of endometrial gland tissue [90–92].

Endometrial glandular organoids derived from patients of endometrial carcinoma and endometriosis have been reported [80, 89, 93]. Endometrial glandular organoids from the endometriotic adenocarcinoma tissue exhibited distinct morphological features [93, 94]. Similar to the morphology of primary tumour, the nuclei of the cells in the organoids showed excessive chromatin, and the epithelium tended to be disorganized. The cells composing the organoids showed invasiveness as one of the key properties of cancer cells. They breached the basement membrane of the organoids and dispersed in the Matrigel as single cells [80]. The morphology of the organoids correlated with the grade of the adenocarcinoma; a clearly defined lumen in the low-grade tumour-derived organoids, but invisible lumen structure with dark and dense morphology in the high-grade tumour-derived organoids. Molecularly, the malignancy-associated marker genes were expressed in the organoids derived from endometrial cancer, and the expression pattern resembled the tumour tissue sample in vivo. The mutated genes in the tumour tissue were retained in the so-derived organoids, even after a long time of in vitro expansion.

Endometriosis is a common gynaecological disease affecting 10% of the women of reproductive age. It is defined by the presence of endometrial-like tissue outside the uterine cavity with associated abdominal pain [95]. Endometrial glandular organoids could be derived from the eutopic endometrium of women with endometriosis but at a much lower proliferation rate and clonogenic efficiency compared with the ones derived from endometrium of healthy women [89, 93]. Morphologically, endometriotic organoids showed unique stratified epithelium that was not present in the wild-type organoids, and the lining cells of the organoid lumen were much thicker than the healthy ones. The cells lining the organoids showed the signs of invasion into the lumen. This feature together with expression of matrix metalloproteinases (MMPs) in the endometriotic organoids reflected the invasive property of the eutopic endometrial tissue in endometriosis in vivo. Molecularly, endometriosis organoids showed a lower level of glycodelin-A expression upon estrogen stimulation compared to the healthy ones, which was the same as the response of the endometriosis tissue biopsies. RNA sequencing data also revealed the expression of endometriosis-specific genes in endometriosis-derived organoids, which coincided with the expression pattern in the tissue biopsy [89].

Even though the endometrial glandular organoid model seems to be a promising tool for the in vitro studies of the endometrial gland, the model has several limitations. As the endometrial glandular organoids possess a basal-out apical-in polarity, the secretions are entrapped in the lumen instead of secreting directly into the culture medium. This feature results in difficulties in collection of the secretory product from the organoids as well as the potential asymmetrical secretion [96]. By either micromanipulation or sequential centrifugation, the intra-organoid fluid could be extracted, which has proved a different metabolite profile compared to the extra-organoid fluid [96]. Inter-patient variation is another obstacle for large-scale application of the endometrial glandular organoid, which may present different expansion ability and even morphology from tissue collected from different patients. In the future, gene manipulation via CRISPR-Cas9 may be a further step for the endometrial glandular organoid models, which would definitely facilitate more studies on the endometrial gland function during pregnancy and in female reproductive disorders.

#### Endometrial assembloids

Many existing 3D culture models including organoids have limitations like use of scaffold, growth factors and difficulties of incorporating multiple cell types. The endometrial 3D mono-spheroids (single cell type) models have been in use for many years to mimic the in vivo environment using non-adherent culture techniques [97]. The first human endometrial heterotypic assembloid was developed using different cell types from the endometrium [98]. The assembloids could capture the unique endometrial epithelial and stromal cell interactions with proper polarity and expression of steroids receptors, and could be used for drug screening. Another heterotypic assembloid model using the endometriotic cell line TZ2 and the human endometrial stromal cell line (T-HESC) was developed to study endometriosis [99]. The model enabled bio-fabrication of endometriosis tissue without the use of scaffold. Since the assembloids do not use scaffold, the generated 3D spheroids can be directly used for bio-fabrication which will be a key factor in future endometrial regeneration and endometrial cell therapy for women with Asherman’s syndrome or thin endometrium. An assembloid model with primary human endometrial cells awaits further work [100]. A 3D spheroid/assembloid model with both endometrial primary epithelial and stromal cells has been developed with bovine endometrial cells [101] implicating the possibility of deriving a similar human model.

## In vitro models for studying trophoblast-endometrium interaction

### Embryo implantation models

The process of implantation is accomplished by the proliferation, differentiation, migration and invasion of the trophoblasts from blastocyst, together with the regulation of the endometrial decidua. To better understand the detailed process of embryo implantation, the incorporation of the blastocyst or blastocyst surrogates with the in vitro 3D endometrial models are widely studied (Table 2), and their applications in association with the endometrial models will be discussed.

**Table 2:** Examples for various implantation models in use

	**Schematic illustration**	**Advantages**	**Physiological process modelling**	**Disadvantages**	**Reference**
Human/mouse blastocyst		- Best recapitulate the morphological features of the blastocyst in vivo	- Embryo implantation	- Ethical issues - Shortage of human blastocysts - Mouse blastocysts have minor difference compared to human	*Lalitkumar *et al*. 2007, Qi, *et al*. 2014, Ruane, *et al*. 2020* [73, 102, 103]
Trophoblast cell line		- Easy to acquire - Relatively easy to set up - Allow the 3D modelling of endoglandular invasion by single extravillous trophoblast cells	- Endoglandular invasion by trophoblast cells	- Immortalised cell line may contain genetic aberration	*Buck, *et al*. 2015* [77]
Trophoblast spheroids		- 3D structure better mimic the physiological implantation process - Can be incorporated with stromal cells and ECM (Matrigel /Fibrin) for 3D invasion studies	- Trophoblast invasion and early placentation	- Mainly derived from cell lines with potential genetic aberrations	*White, *et al*. 1988, You, *et al*. 2019, Akbar, *et al*. 2020* [8, 104, 105]
BAP-EB		- 3D structure highly recapitulate human blastocyst - Able to attach to endometrial epithelial cells for implantation studies	- Embryo implantation in terms of early adhesion, and attachment to endometrial epithelial cells	- High expertise required - Higher cost due to the need of specific differentiation medium	*Lee, *et al*. 2015, Yue, *et al*. 2020* [106, 107]
Blastoids		- Able to differentiate into both embryonic and extra-embryonic lineages	- Post-implantation development (especially in mouse)	- Extremely high expertise required - Highly complicated experimental set-up - Majority of the studies are in mouse but not in human - May associate with ethical issues	*Rivron, *et al*. 2018, Li, *et al*. 2019, Sozen, *et al*. 2019, Yu, *et al*. 2021* [108–111]

#### Human/mouse blastocysts

The most straightforward method to study implantation is the culture of blastocysts, either from human or mouse, on endometrial epithelial cells or 3D endometrial models to mimic early implantation [102, 103, 112, 113]. For example, human blastocysts were cultured on a confluent layer of endometrial epithelial cells to study the impact of mifepristone and levonorgestrel on embryo attachment [73]. In most cases, however, the use of human blastocysts is restrictive due to their limited supply and ethical considerations. Thus, blastocyst surrogates are largely used in implantation studies.

#### Trophoblast spheroids

Trophoblast spheroids, which can be formed either from primary trophoblasts or choriocarcinoma cell lines, were commonly used as blastocyst surrogates for implantation studies. The multicellular spheroids can be generated by culture of trophoblast suspension in a rotation platform or an ultra-low attachment culture plate [8, 104, 114]. These trophoblast spheroids can attach onto the surface of endometrial epithelial cells monolayer in vitro. The attachment rate can be quantified to study the impact of molecular or chemical factors on implantation [105, 114].

To take one step forward, Wang et al. integrated the trophoblast spheroids derived from a choriocarcinoma cell line with their 3D endometrial culture system (Fig. 3C) [72] and demonstrated a significantly higher attachment rate compared to the attachment onto the monolayer of endometrial epithelial cells. Another study established a model investigating the impact of endometrial stromal cells culture on invasion of the trophoblast spheroid through the Matrigel [8]. In the study, endometrial stromal cells at the bottom of the culture plate were covered with Matrigel diluted with the culture medium at the ratio of 1:1 before addition of the trophoblast spheroids. The trophoblast spheroids were able to penetrate and invade into the Matrigel and interact with the underlying endometrial stromal cells. Although the use of carcinoma-derived cell lines for establishment of the trophoblast spheroids overcomes the problem of availability and ethical considerations of human blastocysts, it is questionable whether it could physiologically resemble the human blastocyst and the dynamic implantation process.

#### Human embryonic stem cell-derived trophoblastic spheroids (BAP-EBs)

To overcome the drawbacks of trophoblastic choriocarcinoma spheroids, human embryonic stem cell-derived trophoblastic spheroids were developed as a novel trophoblast surrogate model in 2015 by Lee et al. [106, 107]. Human embryonic stem cell line VAL3 could be differentiated into the trophoblastic cells in specific differentiation medium BAP containing bone morphogenetic protein (BMP) 4, ALK4/5/7 inhibitor and FGF2-signaling inhibitor in mouse embryonic fibroblast-conditioned medium. During the course of BAP-induced differentiation, the expression of the pluripotency marker octamer-binding transcription factor 4 (OCT4) was reduced while that of the trophoblastic markers (Human Leukocyte Antigen-G (HLA-G), β-hCG, Cytokeratin 7 (CK7) and H19) was increased. Moreover, the BAP-treated VAL3 cells showed enhanced capacity of invasion and migration in vitro, consistent with their trophoblastic properties. Embryoid bodies (EB) could be formed by self-aggregation of single-cell culture of VAL3 cells in the AggreWell® plate. BAP treatment converts the EB into a blastocyst-like structure with a cystic structure, termed as BAP-EB. The mRNA profile of BAP-EB resembles that of the trophectoderm of blastocyst before implantation [107]. BAP-EBs attached specifically to the primary human endometrial epithelial cells at the receptive phase but not at the pre-receptive phase. When co-cultured with endometrial stromal cells, BAP-EBs invaded through the stromal cells resembling the human blastocysts during early implantation.

Compared to the classic trophoblast spheroids, BAP-EBs are derived from embryonic stem cells, which allow for long-term culture with an unlimited supply. Moreover, the BAP-EBs selectively attach to the receptive endometrial cells instead of the non-selective attachment ability of the trophoblast spheroids. Morphologically, unlike the multicellular trophoblast spheroids, BAP-EBs possess the blastocoeal cavity-like structure, which resembles the human blastocyst at a more physiological extent. Together, the BAP-EB model may serve as another useful tool for the investigation of early implantation in humans.

#### Blastoid model

Due to the unique developmental plasticity of the naive embryonic stem cells, their capacity to be modelled in vitro has been intensively studied. By exposing the naive human embryonic stem cells to designated differentiation media, they can differentiate into the embryonic and the extraembryonic cell lineages, including the SOX2 positive epiblast-like cells, GATA6 positive hypoblast-like cells and GATA3 positive trophoblast-like cells [108–110, 115, 116]. The first study on establishment of blastocyst-like structures from embryonic stem cells termed ‘blastoid’ was published in 2018 [108]. By culturing in non-adherent microwells, the embryonic stem cells self-aggregated to form spheroids, which were then overlaid with trophoblast stem cells. The resulting human blastocyst-like structure possessed a cyst-like structure surrounded by TE-like cells and a cell aggregate inside the cyst resembling the ICM. Molecularly, the blastoids expressed pluripotency markers (OCT4 and NANOG) and a considerable level of trophoblast marker (Caudal-type Homeobox 2 (CDX2)) in the presence of culture supplement (IL-11, 8Br-cAMP, FGF4 and Transforming Growth Factor β1 (TGFβ1)). Moreover, transfer of the blastoids into pseudo-pregnant female mice induced decidualization of the endometrium of the recipient.

Human blastoids could be derived solely from human embryonic stem cells [110]. The resulting blastoids expressed the marker genes present in human blastocyst including SOX2, SOX17 and GATA3. Single-cell RNA sequencing analysis revealed that the blastoids contained epiblast-like cells, hypoblast-like cells and trophoblast-like cells comparable to the cell clusters reported in human blastocysts. Several studies have established the blastoid models using either human or mouse embryonic stem cells, which all showed functional, molecular and morphological similarities to the blastocysts in vivo [109, 111, 117].

The blastoid model possesses the most physiological structure and morphology resembling the blastocyst in vivo, which is unique for studies on preimplantation embryo development and early pregnancy in vitro. However, the specific culture condition and the high expertise required for the blastoid culture are obstacles for their large-scale application. Another concern is public acceptability of research on blastoids, which could potentially give rise to live birth. That possesses an ethical issue that researchers need to consider.

### Trophoblast migration and invasion models

The migration and invasion of the EVTs play important roles for successful placentation. After implantation, the TE develops into the cytotrophoblastic shell. CTBs within the shell is disrupted and differentiate into multiple clusters of proliferating EVTs, which invade the decidua via the interstitial or the endovascular routes [30]. In the decidua, the EVTs remodel the spiral arteries to low-resistance high-flow vessels to provide sufficient feto-maternal exchange. To better understand the molecular drive of this procedure, several EVT migration and invasion models have been established.

#### EVT migration and invasion models

**Fig. 5:**
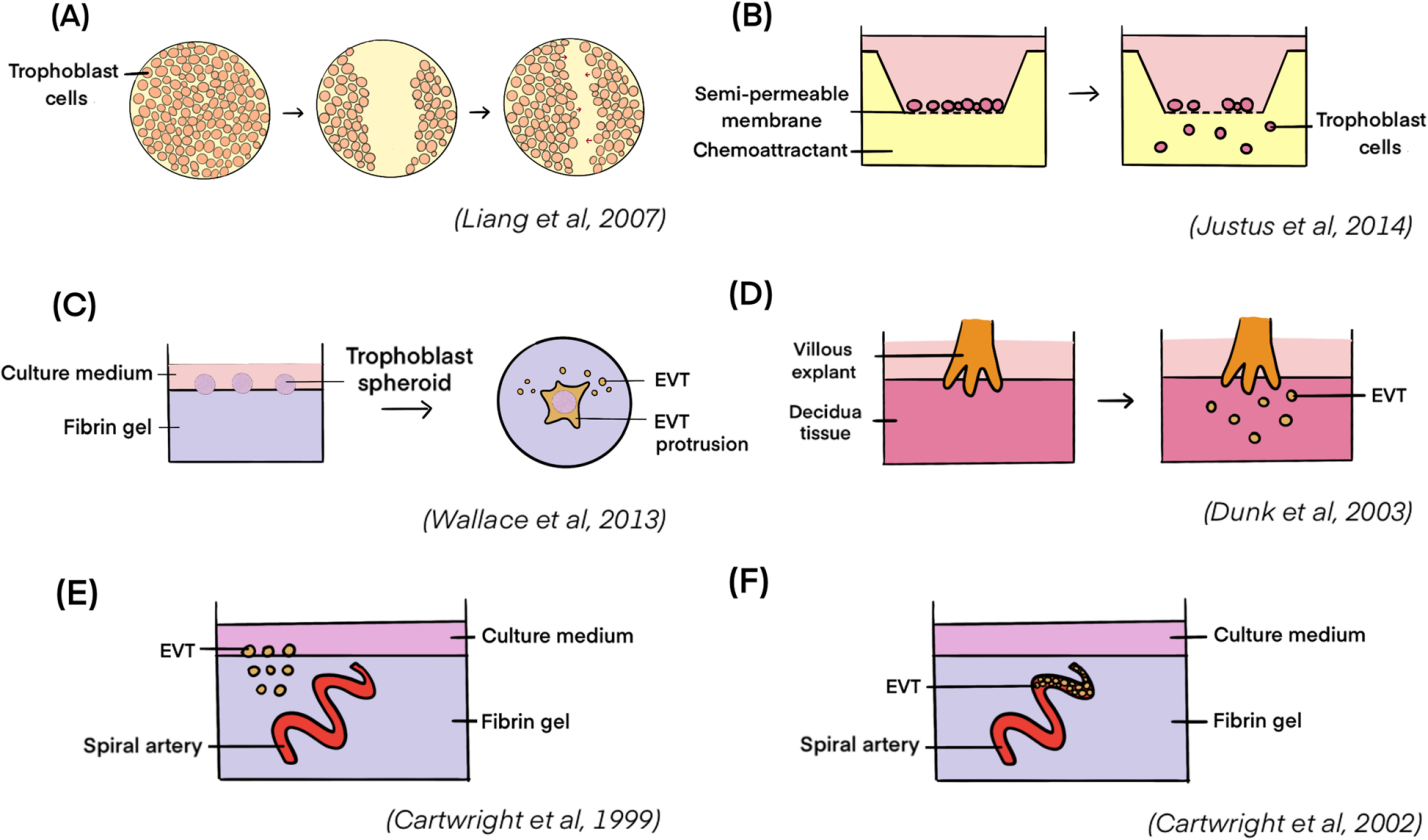
In vitro models/assays for trophoblast invasion studies. **A** Scratch/wound healing assay; **B** Transwell study; **C** Trophoblast invasion/protrusion assay; **D** Villous explant co-cultured with the decidua tissue; **E** Interstitial invasion model of EVT into spiral artery-embedded gel; **F** Endovascular invasion model

For the in vitro studies of EVT migration and invasion, the sources of the EVTs are the choriocarcinoma-derived cell lines (e.g. JEG-3, JAR and HTR-8/SVneo), primary trophoblasts isolated from first-trimester placenta, or villous explants from placenta [118–121]. Several EVT migration and invasion assay models have been established in 2D and 3D formats (Fig. 5). The most common assay of EVT migration is the scratch/wound healing assay (Fig. 5A), which simply studies the migration ability of the cells in a 2D condition [122]. The transwell assay (Fig. 5B) examines the migration capacity of the cells in a 3D setting [123]. In the assay, the cells in a culture insert migrated through a semi-permeable membrane under the drive of the chemoattractant of interest below the insert. The number of migrating cells under different experimental conditions could be easily quantified by histological staining of the cells [124]. To mimic the ECM or endometrial stroma during early pregnancy, the semi-permeable membrane could be coated with Matrigel, which is known as the Matrigel invasion assay [125, 126]. Even though majority of the current trophoblast invasion models do not include the maternal endometrial cells, some of the studies have incorporated the conditioned medium secreted by the pre-cultured endometrial cells to study their impact on trophoblast invasion [127–130]. For example, Godbole et al. established the Matrigel invasion assay by adding the conditioned medium produced from decidualized endometrial stromal cells at the lower chamber of the culture insert, and identified a significantly higher number of JEG-3 cells invading through the Matrigel in comparison to the ones cultured with conditioned medium collected from non-decidualized stromal cells [127]. Alternatively, EVT invasion could be studied by seeding the trophoblast spheroids onto the fibrin gel and quantifying the mean number and length of the invasive protrusions (Fig. 5C) [126, 131].

Both wound healing assay and the transwell assay are easy to experiment thus, widely utilized for the EVT migration studies. However, they lack the physiological environment as only the trophoblasts were involved in the culture system. They cannot fully recapitulate the in vivo condition during placentation. Therefore, a 3D model with maternal tissue may be a further goal to be explored.

#### Maternal-tissue integrated EVT migration and invasion models

The in vitro model depicted in Fig. 5D incorporated the decidua tissue with the co-cultured villous explant to better study trophoblast-decidual invasion in a 3D condition [132]. In the model, the villous explant tissue from first-trimester placenta was placed on top of the decidua tissue from the same donor. EVT columns showing EVT markers HLA-G could be formed at the bottom of the villous tissue penetrating and invading the decidua tissue in vitro. The model allows study of the regulatory factors of trophoblast invasion and migration, such as the stimulatory action of decidua cell-derived growth factor EGF via regulation of the gap junction protein connexin 40 [133].

The remodelling processes of the maternal spiral arteries by the invading EVTs can be studied in vitro by measuring the trophoblast invasion into the endothelial cells [134, 135]. For study of interstitial invasion, spiral arteries were isolated from the myometrium biopsies and embedded in fibrin gel, and fluorescence-tagged trophoblasts were seeded on top of the embedded spiral artery (Fig. 5E). Invasion of the trophoblasts into the fibrin gel occurred after 5 days of culture. For investigation of endovascular invasion, the isolated spiral arteries were perfused with fluorescence-labelled trophoblasts followed by closure of the ends of the spiral arteries (Fig. 5F). The trophoblast-perfused spiral arteries were then immobilized in the fibrin gel and cultured for 3 days. Both models allow the study of the remodelling of the spiral arteries by the invasive EVTs, which serve as suitable models for the pregnancy-associated complications with aberrant spiral artery remodelling, like PE.

Although these models are promising and physiological, they are not widely used due to their disadvantages. First, the availability of high-quality primary tissue sample is limited, and inter-patient variation may lead to inconsistent results. Second, the culture of the primary tissue for a long time is challenging due to tissue necrosis in vitro [126]. The primary CTB cells harvested from first trimester placentae usually show diminished proliferative capacity in vitro. Better EVT migration and invasion model is urgently required, which may allow the participation of the in vitro 3D culture models of endometrium to demonstrate the process more physiologically.

#### Trophoblast organoid model

**Fig. 6:**
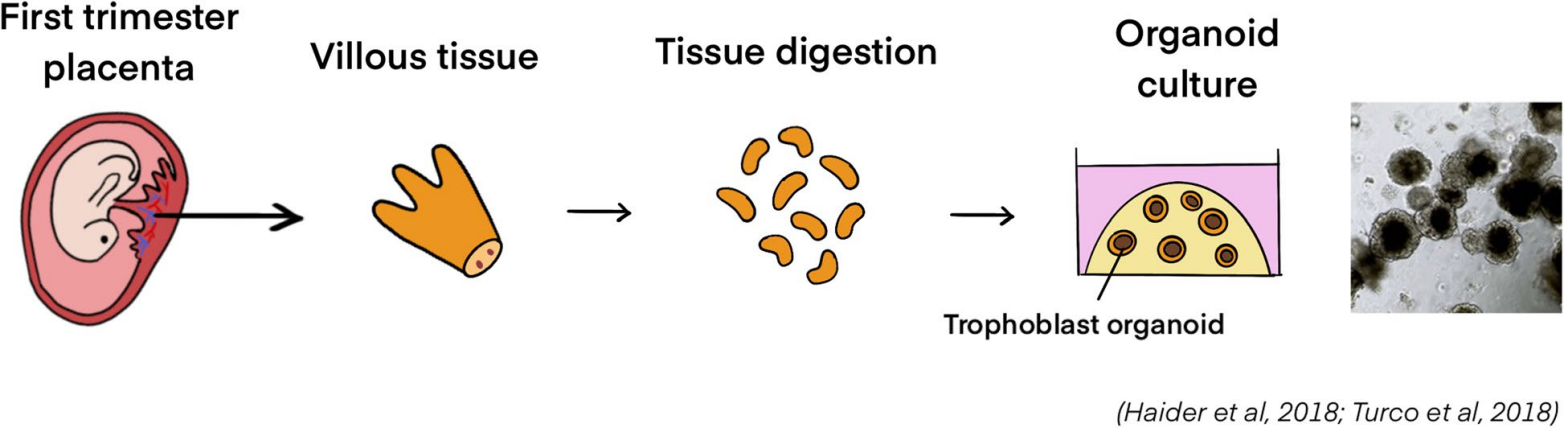
Establishment of trophoblast organoids. Villous tissue collected from first trimester placenta is digested and the trophoblast cells will then be seeded in Matrigel for the formation of trophoblast organoids

Trophoblast organoids have been established as a 3D model for the trophoblast differentiation, invasion and migration studies [136, 137]. Placental villi isolated from the first trimester (6 ~ 9 weeks of gestation) placentae are the primary source of the trophoblast organoids, which can self-organize into 3D organoid structures in 2 ~ 3 weeks after embedding in Matrigel and stimulation/inhibition of specific signaling pathways (Fig. 6). Physiologically, the placental villi developed from the TE of blastocysts are composed of CTBs, which eventually differentiate into the hormone-secreting STBs and the decidua/spiral artery-invading EVTs. The trophoblast organoids expressed several trophoblast markers, including GATA3, KRT7, EGFR, TFAP2A and TFAP2C [136, 137]. Compared to the in vivo condition, the villous cytotrophoblast (VCT) markers Ki67 and TP63, the SCT markers CD46 and CD71 were all expressed in the trophoblast organoids [137].

The trophoblast organoids are genetically stable and show structural, morphological, functional and metabolic similarities to the villi in vivo. When cultured in a specific EVT differentiation medium, the trophoblast organoids were able to differentiate into EVTs that were positive for EVT marker HLA-G. The HLA-G^+^ EVT cells from the organoids exhibited migratory and invasive activities in Matrigel. The trophoblast organoids harbor all functional trophoblast cell types in the human villi, and they also pocess secretory functions of SCT and several placental hormones and peptides, such as hCG, Growth/differentiation Factor 15 (GDF15) and Pappalysin 1 (PAPPA) of SCT under the stimulation of specific ST differentiation medium [137–139].

The co-culture of the trophoblast organoids with the in vitro 3D culture model of endometrium will potentially allow the recapitulation of trophoblast-endometrium interaction during early placentation under a more physiological environment. Yet the further optimization of this model, for example, to reverse the polarity of trophoblast organoids (from basal-out/apical-in to apical-in/basal out), is required in the near future for the better implications into epithelial biology and trophoblast-endometrium interactions studies.

## Future perspectives of the 3D culture models of endometrium for modelling of defective trophoblast-endometrium interaction

**Fig. 7:**
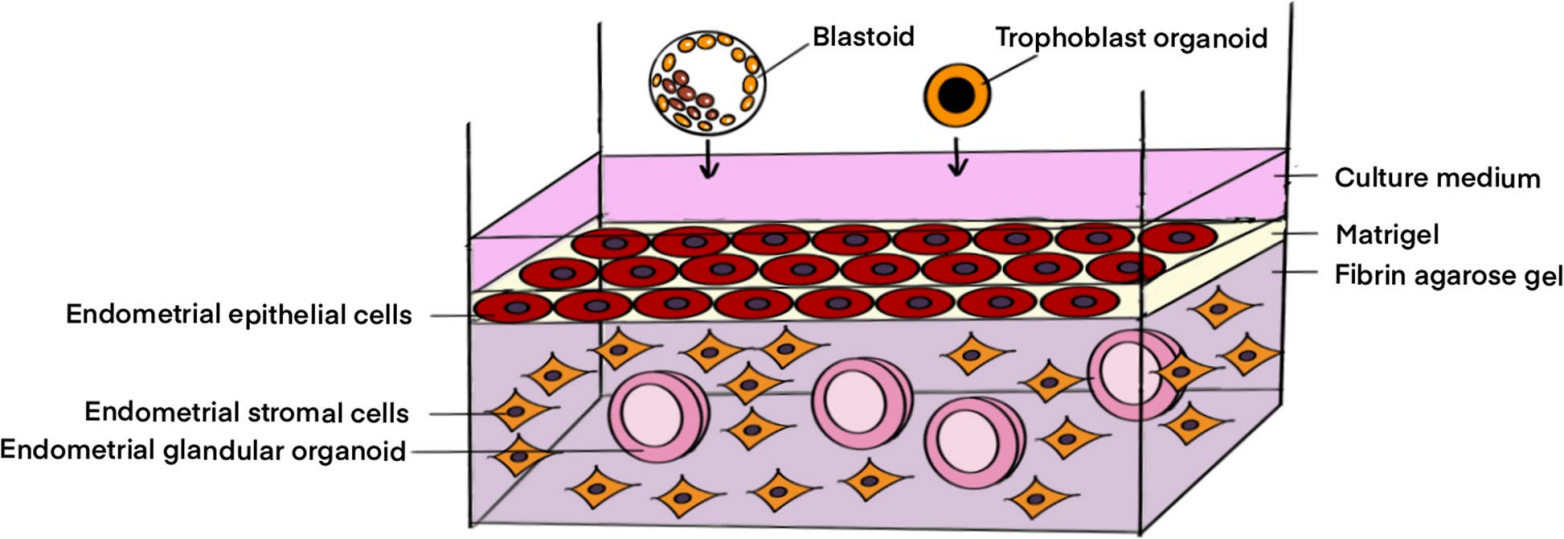
Illustration of a 3D model according to the reviewed studies for trophoblast-endometrium interaction investigation. Combining the currently available 3D endometrial models, a novel 3D model is proposed which integrates endometrial cells including endometrial epithelial cells and stromal cells, endometrial glandular organoids, blastoids and trophoblast organoids, for the purpose of better recapitulation of embryo implantation and placentation

Although a great number of culture models have been established, majority of them are still in their initial stage of application. Nevertheless, they have the potential to mimic both the embryo implantation and the early pregnancy, which would facilitate the investigation of implantation failure or recurrent pregnancy loss and pregnancy complications such as PE. Combining the endometrial glandular organoids/trophoblast organoids/blastoid models with the currently available endometrial models may better recapitulate the physiological events during trophoblast-endometrium interaction, which has rarely been done now. A comprehensive endometrial model, for example the one established by Wang et al. [72] which incoporated endometrial stromal cells and epithelial cells with the ECM. By adding the endometrial glandular organoids into the system, their unique properties, such as the preservation of hormone responsiveness and secretory function, would resemble the physiological condition of the human endometrium for a better recapitulation of the in vivo environment. According to the reviewed studies, we proposed a potentially achievable model for the study of trophoblast-endometrium interaction as depicted in Fig. 7. The model incorporates the endometrial epithelial cells in collagen/Matrigel laying on top of endometrial stromal cells and endometrial glandular organoids-packed in fibrin-agarose gel as the endometrium surrogate, while the blastoids or trophoblast organoids are employed as the blastocyst and early placental villi surrogate, respectively. To further recapitulate the physiological microenvironment, the immune cells and the endothelial cells can be assembled into the system.

By incorporating the cells and organoids derived from patients of interest, the proposed model may facilitate the demonstration and modelling of different diseases that have been known to contribute to infertility and pregnancy failure. For example, normal blastoids may be added to the 3D culture models of endometrium established from the endometrial cells of patients with repeated implantation failure, which hopefully could demonstrate the defective interaction of the trophectoderm onto the endometrial epithelial cells. In terms of recapitulation of early pregnancy and studies of recurrent pregnancy loss, endometrial biopsies could be collected from respective women to establish the 3D culture models with the endometrial glandular cells and stromal cells from the same patients. Trophoblast organoids can be incorporated into the culture system to mimic the placentation process during early pregnancy, which may allow the demonstration of defective interaction during trophoblast-endometrium interaction at cellular and molecular levels. For the study of diseases associated with trophoblast function, such as PE, trophoblast and endometrial glandular organoids may be developed from PE patients and incorporated into the 3D model to study their functional abnormalities.

To establish such a novel endometrial model, however, several hurdles are still present. The selection of the culture medium supporting the co-existence of multiple cell types is the major obstacle for the establishment of in vitro 3D culture models of the endometrium. As the culture condition for the organoid system is comparably complicated due to the requirement of specific growth factors, the optimal culture medium of the culture system would be challenging. Another challenge is the biological variation of clinical samples. Even though the endometrial glandular organoids have been shown with genetical stability for more than six months of culture, their stability and viability when incorporated into the 3D culture model would be unpredicted. Thus, the functional durability of the culture models will still be unknown.

In the next step, the proposed culture model could be further improved by assembling of dynamic microfluidics which allows the accomplishment of organ-on-chips systems [140]. Microfluidics system allows the manipulation of fluids flew into the culture system with multiple cell/tissue compartments incorporated to mimic the blood perfusion in vivo. The incorporation of this microfluidics system into the currently available 3D culture model may further promote the paracrine communications of the cells, enhance the comprehensive functionality, and allow the long-term maintenance of the culture system. By controlling the fluid parameters such as shear force and concentration gradient, the whole culture system could resemble a vascularized tissue or organ, thus bring into the function of endothelial cells which are usually absent in previous 3D models. Meanwhile, the specific shear force that can be manipulated would allow the study of specific cell types and the inter-cellular communications within the system, which may help to model pathophysiological conditions [140–143]. This technique would be especially beneficial for the studies in reproductive tissue, whereby the supplementation of sex hormones into the system may allow to mimic the dynamic hormonal change during the human menstrual cycle in a more physiological setting. It has been shown that under this kind of dynamic environment, the endometrial stromal cells showed a greater extent of decidualization in response to the secretory products from the co-cultured endothelial cells upon the stimulation of designated haemodynamic force [141], which highlighted the potential of this technique for modelling of human endometrium in terms of paracrine inter-cellular communications. Another study that incorporated multiple female reproductive organs/tissue into the microfluidic culture system has simulated the menstrual cycle for 28 days with sustained circulation [143]. In their study, successful follicle development in the ovary tissue has been identified, together with a prominently higher secretion of sex hormones in response to the dynamic flow. That result did not only reveal the possibility for this organ-on-chip culture system to be maintained for a longer period of time, but also showed that the cells tend to function more physiologically under the in vivo condition.

In sum, the combination of these techniques and culture models would allow the modelling of both physiological and pathological conditions during the course of human reproduction, such as implantation, early pregnancy, pregnancy failure and pregnancy-associated disorders, as majority of those conditions possess the features of relatively long duration and the presence of hormone cues, which are yet difficult to be mimicked according to currently available cell culture techniques. That would definitely be a huge breakthrough if such comprehensive, physiological and manipulatable models could be invented for reproductive studies in the future.

## Conclusion

In vitro 2D to 3D models of endometrium are good tools for understanding the molecular mechanism behind embryo implantation and early pregnancy in humans. By introducing the newly established organoid concept, including the endometrial glandular organoids, endometrial assembloids, trophoblast organoids and blastoid model, the in vitro 3D culture models can better recapitulate the trophoblast-endometrium interaction for investigation of the pathophysiology of implantation failure or pregnancy complications such as recurrent pregnancy loss and PE. The outcome of the investigations on patient-derived endometrial organoids/assembloids would enable detection of potential biomarkers and causative factors for early diagnosis and development of novel treatment strategy. Although the integration of these models needs to be optimized, they set the basis for ideal modelling of the endometrium, which would eventually benefit fertility treatment.

## Data Availability

Not applicable.

## Abbreviations

2D: Two dimensional
3D: Three dimensional
BMP: Bone morphogenetic protein
CDH1: Cadherin 1
CDX2: Caudal-type homeobox 2
CK7: Cytokeratin 7
COX-2: Cyclooxygenase-2
CTB: Cytotrophoblast
dNK: Decidual nature killer
EB: Embryoid bodies
ECM: Extracellular matrix
EGF: Epidermal growth factor
ER: Estrogen receptor
EVT: Extravillous cytotrophoblasts
FGF: Fibroblast growth factor
FOXA2: Forkhead Box A2
GDF15: Growth/differentiation factor 15
hCG: Human Chorionic gonadotropin
HGF: Hepatocyte growth factor
HLA-G: Human Leukocyte Antigen-G
ICM: Inner cell mass
IL: Interleukin
LIF: Leukaemia inhibitory factors
MMPs: Matrix metalloproteinases
MUC-1: Mucin-1
OCT: Octamer-binding transcription factor
PAPPA: Pappalysin 1
PAX8: Paired Box 8
PE: Pre-eclamspia
PR: Progesterone receptor
SOX: SRY-Box Transcription Factor
STB: Syncytiotrophoblasts
TE: Trophectoderm
TGF: Transforming Growth Factor
T-HESC: Human Endometrial Stromal Cell Line
VCT: Villous cytotrophoblast
VEGF: Vascular endothelial growth factor
WOI: Window of implantation
